# Structural and Optical Characterization of Pure and Lanthanum Substituted Nd_1–*x*_La_*x*_AlO_3_ Prepared by Sol–Gel Method

**DOI:** 10.1002/open.202400499

**Published:** 2025-04-07

**Authors:** Akash S. Padole, Vikash Singar, Payal Ratnawat, Ansh Dabkara, Dibya Prakash Kar, Nikita Jain, Minal Gupta, Archna Sagdeo, Pankaj R. Sagdeo

**Affiliations:** ^1^ Materials Research Laboratory Department of Physics Indian Institute of Technology Indore Indore Madhya Pradesh 453552 India; ^2^ Department of Mechanical Engineering University of South Carolina Columbia SC 29208 USA; ^3^ Accelerator Physics and Synchrotrons Utilization Division Raja Ramanna Centre for Advanced Technology Indore 452013 India; ^4^ Department of Physics Homi Bhabha National Institute Training School Complex Anushakti Nagar Mumbai 400094 India

**Keywords:** crystal‐field transition, optical absorption spectroscopies, sol–gel synthesis

## Abstract

This work demonstrates the tuning of crystal‐field parameters of Nd rare Earth ion in NdAlO_3_ through lanthanum (La) substitution; for this purpose, this work has prepared the polycrystalline samples of pure and La‐substituted NdAlO_3_ using the sol–gel synthesis method. The purity of the phase of Nd_1–*x*_La_*x*_AlO_3_ (*x* = 0, 0.1, 0.2, 0.3, 0.4, 0.5) samples has been confirmed via X‐ray diffraction measurements. This work found that with La substitution, the lattice parameters systematically increase. The values of lattice parameters “a” and “b” changed from 5.315 Å (*x* = 0) to 5.339 Å (*x* = 0.5). The lattice parameter “c” also increased from 12.926 to 13.025 Å in these samples. Additionally, the Nd/La—O bond length increased from 2.419 to 2.469 Å. The Nd—O—Nd bond angle shifted from 169.7° to 171.4°, whereas the Al—O—Al bond angle varies from 165.5° to 167.8° and the value of the tolerance factor from 0.9640 to 0.9700, indicating improvement in the structural distortion. The crystal‐field transitions are investigated using optical absorption spectroscopy; this work found that La‐substitution leads to changes in the transition peak 4F_9/2_, which is shifted from 676.99 nm for sample (*x* = 0) to 675.44 nm for sample (*x* = 0.5).

## Graphical abstract

The variations in crystal‐field splitting parameter Δ for the pure and elongated polyhedra. The present studies offer the new methodology to tune crystal‐field energy by means of lattice distortion. The present results are useful for fine tuning of laser energy.

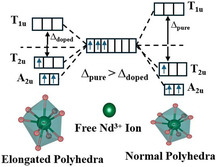

## 1 Introduction

The perovskite family of materials has gained significant attention due to its unique structure and diverse range of applications.^[^ ^1^, ^2^ ^]^ Most of the elements from the periodic table can combine to form ABO_3_ compounds with the perovskite structure.^[^ ^3^, ^4^ ^]^ This versatility of elements capable of forming perovskite structures and creating cation or anion‐deficient configurations results in an extensive range of physical and chemical properties. Consequently, ABO_3_ perovskite‐based oxide systems are applicable in various fields, including energy storage devices,^[^ ^5^ ^]^ electrode and electrolyte material in fuel cells,^[^ ^6^ ^]^ magnetism,^[^ ^7^ ^]^ ferroelectrics, large dielectric,^[^ ^8^ ^]^ oxygen reduction reaction, etc.^[^ ^9^ ^]^ Among the various perovskite materials, rare Earth aluminates, particularly NdAlO_3_, attract special interest due to the luminescence properties of Nd^3+^ ions, which are promising to develop high‐power laser systems.^[^ ^10^ ^]^ Additionally, NdAlO_3_ finds applications as a phosphor for fluorescent lighting, optical amplifiers, and diffusion barriers in solid oxide fuel cells.^[^ ^10^, ^11^ ^]^ These unique characteristics place NdAlO_3_ at the forefront of research. The synthesis method used for materials plays an important role, as it directly affects the final product's distribution, crystallinity, purity, and morphology. Over the years, several methods have emerged for synthesizing NdAlO_3_, including sol–gel synthesis, solid‐state reactions, combustion methods, and chemical vapor deposition.^[^ ^10^, ^12^, ^13^, ^14^ ^]^ Among these, the sol–gel synthesis is particularly effective in achieving the high chemical uniformity and allows for the precise adjustments of the parameters that influence particle nucleation and growth.^[^ ^15^, ^16^ ^]^ This process typically requires lower temperatures than traditional synthesis techniques, reducing energy consumption.^[^ ^15^, ^16^, ^17^ ^]^ Additionally, the sol–gel process effectively substitutes dopants in a compound, ensuring a uniform distribution throughout the material.^[^ ^15^, ^16^, ^17^, ^18^, ^19^, ^20^ ^]^

It is known that the Neodymium ion (Nd) shows crystal‐field transitions, which are governed by the electrostatic interaction between Nd ion and surrounding oxygen anions, leading to the splitting of their f‐orbitals.^[^ ^21^, ^22^, ^23^, ^24^ ^]^ This splitting can be explained by crystal‐field theory, which describes the lifting of the degeneracy of orbital states due to the internal static electric field, also referred to as crystal field or internal stark effect.^[^ ^21^, ^25^ ^]^ This static electric field is produced by surrounding anions/cations (charges), and such a static electric field can be tuned with the changes in bond lengths of corresponding cation–cation or cation–anion pairs (d/f orbital metal ion and legends). The abovementioned crystal‐field transition energies may be probed using optical absorption spectroscopy, and any finite change in the bond length of Nd—O bond length may also be probed.^[^ ^25^, ^26^ ^]^ Some research groups have confirmed that the crystal‐field transition of rare Earth elements in garnet and aluminum oxide materials substituted with Nd ions results in a significant crystal‐field splitting because of Nd^3+^ having three unpaired f‐orbital electrons. These crystal‐field transitions are crucial for the energy of laser radiation in the case of laser, where Nd^3+^ serves as an active lasing element.^[^ ^21^, ^22^, ^23^, ^24^, ^27^ ^]^

It is known that the crystal‐field splitting parameters are essentially controlled by near‐neighbor ions through electrostatic interaction involving the numerical value of charges and position coordinates i.e., bond distances. It is important to mention here that the experimental verifications of the same are absent in the available literature, where the present manuscript is one of the experimental efforts to show the correlation between the crystal‐field parameters and near‐neighbor electrostatic interactions. This study investigated the effect of La substitution on the crystal‐field energy of the f‐orbital in Nd^3+^ ions of NdAlO_3_. For these, we chose La cation as a substituent. It is important here to note that the La ion does not show any crystal‐field transition of its own due to the absence of f orbital electrons in its outermost orbit. The samples Nd_1–*x*_La_*x*_AlO_3_ (where *x* = 0, 0.1, 0.2, 0.3, 0.4, 0.5) were prepared using the sol–gel method. X‐ray diffraction (XRD) and Rietveld refinement confirmed the rhombohedral phase with systematic increments in lattice parameters and bond lengths. Optical absorption spectroscopy shows a shift in Nd‐related crystal‐field transitions to shorter wavelengths as bond lengths vary. It also shows a reduced energy difference between corresponding absorption peaks, highlighting changes in crystal‐field splitting.

## 2 Experimental Section

### 2.1 Sample Synthesis via Sol–Gel Method

The sol–gel method was used to prepare compositions of La‐substituted NdAlO_3_ samples. Specifically, for the preparation of compositions Nd_1–*x*_La_*x*_AlO_3_ (where *x* = 0, 0.1, 0.2, 0.3, 0.4, 0.5), the required proportion of metal nitrate, citric acid, and ethylene glycol was measured according to the following Reaction (1).

(1) $$\text{Nd }{\left({\text{NO}}_{3}\right)}_{3}\cdot 6H_{2}\text{O }+\text{La}{\left({\text{NO}}_{3}\right)}_{3}\cdot 6H_{2}O+\text{Al}{\left({\text{NO}}_{3}\right)}_{3}\cdot 9H_{2}O\to {\text{Nd}}_{1-x}{\text{La}}_{x}{\text{AlO}}_{3}$$

An aqueous solution of NdAlO_3_ and La‐substituted NdAlO_3_ was prepared by dissolving neodymium (III) nitrate hexahydrate Nd(NO_3_)_3_·6H_2_O (purity 99.99%, Thermofisher), lanthanum (III) nitrate hexahydrate La(NO_3_)_3_·6H_2_O (purity 99.99%, Thermofisher) and aluminum (III) nitrate nonahydrate Al(NO_3_)_3_·9H_2_O (purity 99.99%, Thermofisher) in deionized water (DI water). The compounds were mixed in stoichiometric amounts with continuous stirring at a temperature of 373 K. Then, citric acid C_6_H_8_O_7_ (purity 99.5%, Loba) and ethylene glycol C_2_H_6_O_2_ (purity 99%, Loba) were added to the transparent solution as a catalyst and gelation agent, respectively.

**Figure 1:**
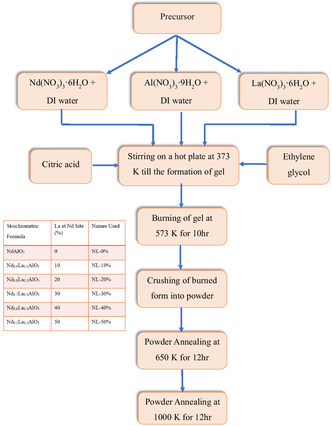
A flowchart of the sol–gel synthesis process with a nomenclature table.

The solution was heated and stirred for about 50 min until a gel formed. The gel was then placed in a muffle furnace for an additional 12 h at 650 K. The powder was again grounded and subsequently kept in the furnace for another 12 h at 1000 K. The final product was obtained in a homogenous dry powder form, and all characterizations were performed. The flowchart outlining the proposed methods for the synthesis is illustrated in **Figure** **1**.

### 2.2 Structural Characterization

#### 2.2.1 XRD and Rietveld Refinements

The purity of the synthesized samples was confirmed by performing XRD measurements using Bruker D8 diffractometer. The following parameters were employed during the XRD measurement at room temperature: tube current, 40 mA; tube voltage 40 kV; scan range, 20 to 80° (2*θ*); wavelength of 1.5406 Å (Cu‐k alpha); and a step size of 0.00203°. The FullProf software suite was used for the Rietveld refinement method to obtain crystallographic information.^[^ ^28^, ^29^ ^]^ This modern crystallographic approach helped to understand the La‐substituted NdAlO_3_ system in terms of crystal distortion. Crystallographic information, including lattice parameters, bond lengths, and bond angles, was obtained through the Visualization for Electronic and Structural Analysis (VESTA) software.^[^ ^29^, ^30^, ^31^, ^32^ ^]^

#### 2.2.2 Optical Absorption Spectroscopy

The optical absorption spectra of the parent and La‐substituted samples were collected at room temperature using an Agilent Technologies Cary 60 high‐precision ultraviolet visible diffuse reflectance spectrophotometer over a wavelength range of 200 to 900 nm with the scan rate of 60 nm min^−1^.

## 3 Result and Discussion

The synthesis of La‐substituted NdAlO_3_ (Nd_1–*x*_La_*x*_AlO_3_) was successfully achieved using the sol–gel method. The mechanism of this method is explained below.

### 3.1 Mechanism of Sol–Gel Synthesis

The sol–gel process involves the transition from a sol (liquid) to a gel (semisolid) through a series of chemical reactions. In the case of synthesis of the Nd_1–*x*_La_*x*_AlO_3_ compound, we used a precursors neodymium (III) nitrate hexahydrate [Nd(NO_3_)_3_·6H_2_O], lanthanum (III) nitrate hexahydrate [La(NO_3_)_3_·6H_2_O], and aluminum (III) nitrate nonahydrate [Al(NO_3_)_3_·9H_2_O], which participate in the following reactions and process to formation of the desired compound.

#### 3.1.1 Hydrolysis Reaction

Initially, the metal nitrates (neodymium (III), lanthanum (III), and aluminum (III) nitrates) dissolve in deionized water (DI water), and hydrolysis occurs. When the nitrate precursors are dissolved in DI water, they break down into their specific metal and nitrate ions, which subsequently interact with water molecules to create hydroxyl complexes.^[^ ^15^, ^19^, ^33^ ^]^ This can be simplified as a hydrolysis reaction (2).

(2) $$M{\left(\text{NO}3\right)}_{n}+H_{2}\text{O }\to \text{ M}{\left(\text{OH}\right)}_{n}+{\text{HNO}}_{3}$$

In this context, M refers to the metal cations (Nd^3+^, La^3+^, Al^3+^), and this step allows precise control of the solid phase precipitation, resulting in uniform particle sizes and morphologies.

##### 3.1.1.1 Chelation and Gelation

**Figure 2:**
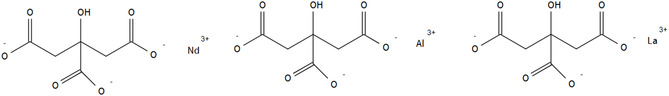
Citrate compounds have negative charges of oxygen and metal cations.

After hydrolysis, the hydroxyl complexes interact with citric acid (C_6_H_8_O_7_), acting as a chelating agent. This interaction allows the citric acid to attach to the metal ions, helping their separation and promoting even distribution throughout the solution.^[^ ^16^, ^33^, ^34^ ^]^ In **Figure** **2**, the structure of the metal citrate compound is shown, and the chelation reaction can be described as reaction (3).

(3) $$M{\left(\text{OH}\right)}_{n}\;+C_{6}H_{8}O_{7}\to M{\left(\text{Citrate}\right)}_{n}+\text{by}\text{-}\text{products}$$

Ethylene glycol acts as a gelation agent, affecting the solution's viscosity, and helps properly mix components, which is crucial for gel creation. When the solution is heated, the increased thermal energy encourages additional polycondensation of the metal‐organic complexes, generating a gel.^[^ ^17^, ^33^, ^34^ ^]^ These processes are described by the following reaction (4).

(4) $$M{\left(\text{Citrate}\right)}_{n}+\text{Heat}\to \text{Gel structure}+\text{gases}$$

This gel formation is important because it creates a 3D network including metal oxides.

##### 3.1.1.2 Annealing Process

After the gel has formed, the gel is annealed at elevated temperatures for extended periods. During this stage, the organic components decompose, and crystalline Nd_1–*x*_La_*x*_AlO_3_ phases are formed. This process is described in the reaction (5).

(5) $$\text{Dry gel}\to {\text{Nd}}_{1-x}{\text{La}}_{x}{\text{AlO}}_{3}+\text{gases/volatile by}\text{-}\text{products}$$

The annealing process was conducted at a temperature of 650 K, effectively removing organic substances and improving the crystallinity of the Nd_1–*x*_La_*x*_AlO_3_ compound. Following this, the powder was re‐grounded to achieve a uniform particle size. An additional annealing step was performed for 12 h at 1000 K, which further promoted the crystallization process and improved the structural coherency and particle size of materials.

### 3.2 Structural Characterization Using XRD

**Figure 3:**
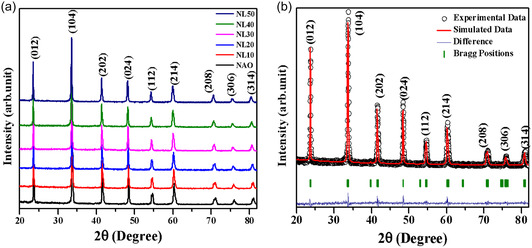
a) XRD data of pure and La‐substituted NdAlO_3_ system. b) Rietveld refinement pattern for La; NdAlO_3_ using space group R‐3c.

The phase purity of pure and La‐substituted NdAlO_3_ samples has been verified using the XRD method. The obtained diffraction data is shown in **Figure** **3**a. All XRD peaks of pure and La‐substituted NdAlO_3_ samples matched well, and no impurity peak was observed. Further, the Rietveld refinement has been carried out by considering the R‐3c space group (167) for the Pure and La‐substituted NdAlO_3_ samples.^[^ ^25^, ^35^ ^]^ Figure 3b shows the representative Rietveld‐refined data of 20% La (Nd_0.8_La_0.2_AlO_3_) substituted sample.

**Figure 4:**
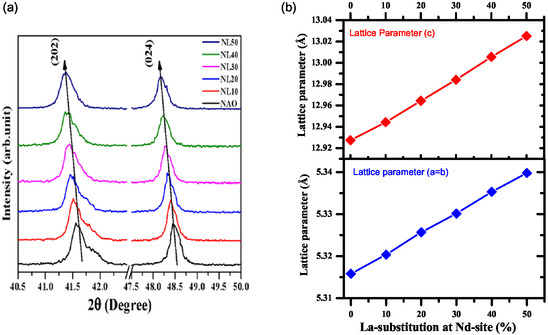
a) Magnified view of XRD of La‐substituted NdAlO_3_. b) Variation of the lattice parameter.

The XRD data clearly shows that no impurity peaks are present. Also, looking into **Figure** **4**a, a systematic decrement in 2*θ* positions of the peaks has been observed, which suggests an increase in the lattice parameters after the La‐substitution. This may be due to the higher value of the ionic radius of La^+3^ (1.216 Å) compared to that of Nd^+3^ (1.163 Å).^[^ ^36^ ^]^ In addition, the lattice parameters were analyzed using Rietveld refinement, and their variation with La substitution is shown in Figure 4b. For the pure sample, the lattice parameters are 5.315 Å for both “a” and “b” and 12.926 Å for “c.” When La is substituted at *x* = 0.5, these lattice parameters increase to 5.339 Å for “a” and “b” and 13.025 Å for “c.” These changes reflected the impact of a larger ionic radius of the La substituent. Additionally, the Nd/La—O bond length was found to be increased from 2.419 to 2.469 Å. The Nd—O—Nd bond angle shifted from 169.7° to 171.4°, whereas the Al—O—Al bond angle varied from 165.50 to 167.80, and the value of the tolerance factor was from 0.9640 to 0.9700, indicating improvement in the structural distortion. To extract the value of bond length and bond angle, etc., from refined structure, the VESTA software has been used.^[^ ^30^ ^]^

**Figure 5:**
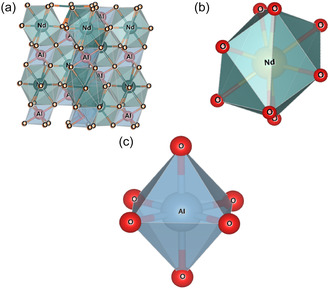
a) Polyhedra structure of La‐doped NdAlO_3_. b) NdO_9_ polyhedron. c) AlO_6_ octahedra structure.

The numerical values of some of the refined parameters and bond lengths are provided in **Table** **1**. Overall, the lattice parameters, bond lengths (Nd/La—O(2)), and cell volume systematically increase with La substitution. The tolerance factor^[^ ^37^ ^]^ (*t*) was calculated to estimate the lattice distortion in the La‐substituted NdAlO_3_ compound, and calculations were performed using the following Equation (6).

(6) $$t=\frac{<Nd[chemistry single bond solid line]O>}{\sqrt{2}<Al[chemistry single bond solid line]O>}$$

where <Nd—O> and <Al—O> denote the average bond lengths between Nd/La—O and Al—O, respectively. Substituting the La ion results in an increase in the value of “*t*,” which is summarized in Table 1. The increasing value of “*t*” indicates a decrease in lattice distortion with La substitution, suggesting a structural improvement. For an ideal perovskite structure, the Al/Nd—O—Al/Nd bond angle should be 180°. The Nd—O(12) polyhedra of La‐doped NdAlO_3_ is shown in **Figure** **5**a. The crystal structure arrangement within the La‐substituted NdAlO_3_ is shown in Figure 5b. It has been observed that out of nine R—O bonds, three R—O(1) bonds are positioned out‐of‐plane with the same bond length, whereas the remaining six R—O(2) bonds are positioned in‐plane and have equal bond lengths. In addition, uniform bond lengths of Al—O bonds in AlO_6_ octahedra are shown in Figure 5c, which indicates that the aluminum ions are situated in an octahedral environment within the octahedral structure.

**Table 1:** Variation of Wyckoff positions and crystallographic information.

Name			NAO	NL‐10%	NL‐20%	NL‐30%	NL‐40%	NL‐50%
Lattice parameter	*a* = *b* [Å]		5.315 (1)	5.320 (1)	5.325 (1)	5.330 (1)	5.335 (2)	5.339 (2)
	*c* [Å]		12.926 (6)	12.944 (6)	12.964 (6)	12.984 (7)	13.005 (8)	13.025 (8)
	*α* = *β*, *γ*		90°, 120°	90°, 120°	90°, 120°	90°, 120°	90°, 120°	90°, 120°
Cell volume	V [Å^3^]		316.364	317.314	318.428	319.451	320.611	321.633
Wyckoff positions	Nd/La	*x*	0	0	0	0	0	0
		*y*	0	0	0	0	0	0
		*z*	0.25	0.25	0.25	0.25	0.25	0.25
	Al	*x*	0	0	0	0	0	0
		*y*	0	0	0	0	0	0
		*z*	0	0	0	0	0	0
	O	*x*	0.5449	0.5447	0.5408	0.5375	0.5379	0.5377
		*y*	0	0	0	0	0	0
		*Z*	0.25	0.25	0.25	0.25	0.25	0.25
Bond angle [°]	Al—O—Al		165.5 (5)	165.5 (5)	166.8 (6)	167.9 (7)	167.7 (7)	167.8 (6)
	Nd/La—O—Nd/La		169.7 (4)	169.7 (4)	170.6 (4)	171.4 (5)	171.3 (5)	171.4 (4)
Bond length [Å]	Al—O		1.8901 (11)	1.8918 (10)	1.8915 (11)	1.8916 (11)	1.8941 (12)	1.8960 (10)
	Nd—O(1)		2.419 (9)	2.422 (9)	2.446 (10)	2.465 (11)	2.465 (11)	2.469 (9)
	Nd—O(2)		2.6559 (8)	2.6589 (7)	2.6607 (8)	2.6628 (8)	2.6667 (9)	2.6701 (7)
Tolerance factor “*t*”			0.9640	0.9639	0.9681	0.9707	0.9705	0.9700
Octahedral tilt φ			8.840	8.802	8.044	7.41	7.47	7.44

The observed distortion within the AlO_6_ octahedra, along with the variation in the Al—O—Al bond angle, indicates notable elongation when compared to the parent NdAlO_3_. According to the glazer notations, the rhombohedral (R‐3c) structure exhibits the a^−^a^−^a^−^ tilt rotation.^[^ ^35^ ^]^ Due to this tilting, distortion occurs in the structure, and this specific tilting results from the rotation of the oxygen anion octahedra found in R‐3c perovskite materials. The following Equation (7) can be used to find the tilt angle.^[^ ^37^, ^38^ ^]^

(7) $$\varphi =tan^{-1}\left(2\sqrt{3}\left(x\left(o\right)-\frac{1}{2}\right)\right)$$

In the above Equation (7), the x(o) represent the position of oxygen in octahedra (AlO_6_) geometry and the value of the tilt angle (φ) for different substitutions of La cation, which are tabulated in Table 1. As the substitution of La cation increases, that octahedral tilt decreases, causing less distortion in the compound.

### 3.3 OAS

**Figure 6:**
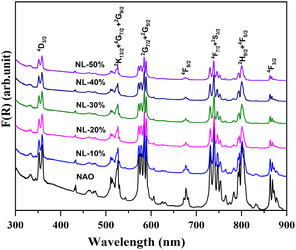
Optical absorption spectra of La‐substituted NdAlO_3_ showing crystal‐field transitions.

**Figure 7:**
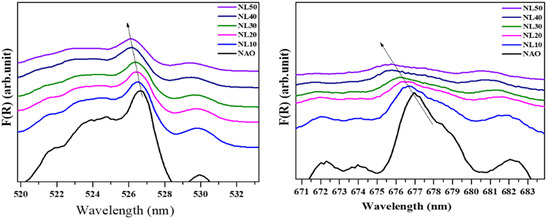
Magnified view of crystal‐field transitions tuned by the La incorporation in the NdAlO_3_ host lattice.

Optical absorption spectroscopy (OAS) helps us to understand the crystal‐field transition associated with the f‐orbitals of the Nd_1–*x*_La_*x*_AlO_3_ sample.^[^ ^25^ ^]^ In this study, we have used OAS in diffuse reflectance mode on prepared Nd_1–*x*_La_*x*_AlO_3_ powder, and it covers a wavelength range from 200 to 900 nm. The resulting optical absorption spectra in **Figure** **6** show a seven distinct crystal‐field transition from the ^4^F_9/2_ state to various states of Nd (neodymium). In this compound, Nd exists in a +3‐oxidation state with an electronic configuration Nd^+3^ (Xe, 4f^3^ 5d^0^), and this configuration allows for f‐f transition due to the presence of f‐orbital electrons, which are governed by the optical transition rule.^[^ ^35^, ^39^ ^]^ In contrast, La (lanthanum) is in a +3 oxidation state with an electronic configuration La^+3^(Xe, 4f^0^ 5d^0^) and does not contain f orbital electrons, so it does not participate in the f‐f transition.^[^ ^39^, ^40^, ^41^ ^]^ However, substituting the La ion into the NdAlO_3_ compound affects the crystal‐field splitting of the Nd ion. As the percentage of La ions increased, we observed a peak shift toward the shorter wavelengths in the associated spectra in **Figure** **7**. The transition peak for the 4F_9/2_ shifted from 676.99 nm for *x* = 0 to 675.44 nm for *x* = 0.5. This observation suggests that the substitution affects the electrostatic interactions between the Nd^+3^ ion and surrounding aluminum (Al^+3^) and oxygen (O^−2^) ions.

Electrostatic interactions quantify the potential energy between the central metal ion (in the present case, Nd^+3^ ion) and surrounding ions (in the present case, O^−2^ and Al^+3^ ions). This energy is influenced by their charge interactions, which is described by Equation (8) of the Coulombic force.^[^ ^21^ ^]^

(8) $$E=K\sum_{i=1}^{n}\frac{q_{i}}{r_{i}}$$

Illustrate that the energy (*E*) depends upon the surrounding charges ($q_{i})$of the Nd^+3^ ion and their respective distance (*r*_*i*_). Variation in the Nd/La—O bond lengths is expected to influence the electrostatic interactions and hence corresponding splitting between the f‐orbitals due to the crystal‐field environment, that is, due to the variation in bond length.^[^ ^42^, ^43^ ^]^

In our study, we calculated an energy difference between two adjacent absorption peaks as mentioned in **Table** **2**, which are represented by the electronic transition between two energy levels at around ≈1.4276 eV and at ≈1.4358 eV. We selected spectra with the same composition of La ions to calculate the energy difference between two corresponding peaks. The resultant calculated data of two corresponding energy peaks, *E*1 and *E*2, which lie in the wavelength range of 800 to 900 nm with the differences (Δ*E* = *E*2–*E*1) spectra, are tabulated in Table 2.

**Table 2:** Variation of transition energy and bond length.

Nd_1–*x*_La_*x*_AlO_3_	Bondlength [Å]	Energy(E_1_) [eV]	Energy(E_2_) [eV]	Energy difference [meV]
La‐0%	2.6559(8)	1.4276	1.4358	8.2
La‐10%	2.6589(7)	1.4281	1.4361	8.0
La‐20%	2.6607(8)	1.4284	1.4361	7.7
La‐30%	2.6628(8)	1.4291	1.4363	7.2
La‐40%	2.6667(9)	1.4301	1.4368	6.7
La‐50%	2.6701(7)	1.4309	1.4369	6.0

**Figure 8:**
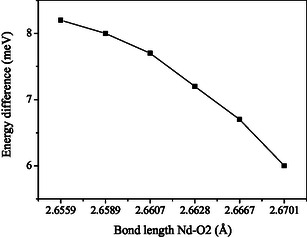
Energy difference (Δ*E* = *E*_2_−*E*_1_) versus bond length (Nd/La—O_2_).

Furthermore, **Figure** **8** shows the variation of the above‐mentioned energy difference ΔE as a function of Nd/La—O bond length. As the La concentration increases on the Nd site in Nd_1–*x*_La_*x*_AlO_3_, the energy difference corresponding to the nearest two peaks decreases, as shown in Table 2. This reduction in energy differences corresponding to the nearest two peaks is associated with bond distances.

### 3.4 Theoretical Discussions

The complex energy level schemes of Nd^3+^ ions within the crystal arise from the interactions between these ions and their surrounding environment, corresponding with the 4f^3^ electron configuration. To calculate these energy levels, such as trivalent ions of the lanthanide series, these can be calculated by diagonalizing the energy matrix. The energy eigenvalues and eigenvectors corresponding to the crystal‐field levels can be obtained by diagonalizing a combined atomic and crystal‐field energy matrix. This process involves evaluating the expectation value of the parametric Hamiltonian, as expressed in Equation (9).^[^ ^44^ ^]^

(9) $$H=H_{A}+H_{\text{CF}}$$

In Equation (9), *H*_A_ stands for the atomic Hamiltonian, while *H*_CF_ is the crystal‐field interaction Hamiltonian. The atomic Hamiltonian can be expressed by Equation (10).^[^ ^45^ ^]^

(10) $$\begin{array}{c} H_{A}=E_{\text{avg}}+\sum_{k=2,4,6}F^{k}f_{k}+\xi A_{\text{SO}}+\alpha L\left(L+1\right)+\beta G\left(G_{2}\right)+\gamma G(R_{7})\\ +\sum_{i=2,3,4,5,6,7,8}T^{i}t_{i}+\sum_{j=0,2,4}M^{j}m_{j}+\sum_{k=2,4,6}P^{k}p_{k} \end{array}$$

Equation (10) includes various operators and parameters representing distinct perturbative terms in the atomic Hamiltonian. The parameter *E*_avg_ represents the spherically symmetric component of the total atomic energy.^[^ ^45^ ^]^ The term $\sum_{k=2,4,6}F^{k}f_{k}$ accounts for the electrostatic repulsion between the 4$f^{n}$ configuration that is described by the term involving *F* ^*k*^ parameters (electrostatic radial integrals) and the *f*_*k*_ (angular part of the electrostatic interaction). The term $\xi A_{\text{SO}}$ describes the interaction between the spin and orbital motion of electrons with *ξ* and *A*_SO_ outlining the radial and angular elements of the spin‐orbit coupling interaction, respectively. The expression $\alpha L\left(L+1\right)+\beta G\left(G_{2}\right)+\gamma G\left(R_{7}\right)$ define as the two‐particle correction term, where the parameters *α*, *β*, and *γ* account for radial interactions and G(G2) and G(R7) are Casimir's operators for the groups G2 and R7, respectively.^[^ ^45^ ^]^ The three‐body interactions in Equation (10) are represented by the following.

(11) $$\sum_{i=2,3,4,5,6,7,8}T^{i}t_{i}$$

where $T^{i}$ is the parameter, and *t*_*i*_ is an operator and the term

(12) $$\sum_{j=0,2,4}M^{j}m_{j}$$

describes magnetically correlated corrections due to electron interactions, with parameters *m*_*j*_ are the operators and *M* ^*j*^ known as Marvin integrals.^[^ ^45^ ^]^ Finally, the electrostatic correlated spin‐orbit interactions are described by the following.

(13) $$\sum_{k=2,4,6}P^{k}p_{k}$$

with *p*_*k*_ as the operators and *P* ^*k*^ as the parameters.^[^ ^45^ ^]^ Crystal‐field interaction involves the distortion of a rare earth ion (Nd^3+^) electron cloud due to surrounding ligands (Al^+3^ and O^−2^). These ligands interact with the ion through electrostatic forces, disrupting its spherical symmetry and causing a split in the ion's energy levels. This splitting results from varying interactions between the electrons and the ligands, leading to crystal‐field splitting. The crystal‐field Hamiltonian, denoted as *H*_CF_, is defined by Equation (14).^[^ ^46^ ^]^

(14) $$H_{\text{CF}}=-e\sum_{i=1}^{n}V\left(r_{i}\right)$$

In Equation (14), the term *V*(*r*_*i*_) indicates the potential experienced by an electron, which is defined by its position vector *r*_*i*_. The summation is performed for all electrons in the system, where *i* ranges from 1 to *n*. The 4$f^{n}$ electrons are influenced by crystal‐field perturbation through a time‐independent charge distribution *ρ*(*R*). The crystal‐field potential can be represented by Equation (15).^[^ ^46^ ^]^

(15) $$V\left(r_{i}\right)=\int \frac{\rho \left(R\right)}{\left|R-r_{i}\right|}d\tau$$

In Equation (15), the term *ρ*(*R*) represents the charge distribution, and $\left|R-r_{i}\right|$ describes the distance between two charged quantities. According to Equation (14) and (15), we expect the potential energy$V\left(r_{i}\right)$ associated with the position vector in La‐doped NdAlO_3_ to follow the pattern described by the inverse function of the position vector/bond length, which aligns with the experimental observations, that is, with a bond length increases, the electrostatic interactions between the neighboring Nd^3+^ ions and oxygen anions weaken (see Table 1 for numerical values). This weakening of interactions may account for the reduced energy splitting due to the change in the crystal‐field environment in the La‐substituted NdAlO_3_.

## 4 Conclusion

In conclusion, we successfully synthesized single‐phase polycrystalline samples of La‐substituted NdAlO_3_ using the sol–gel method. The structural analysis conducted through XRD confirmed the structural improvement due to La ion substituent, with lattice parameters increasing from the pure sample to the doped sample. Additionally, we observed an increase in the Nd/La—O bond length alongside a shift in the bond angle, reflecting the influence of the larger ionic size of the lanthanum ions. Our optical absorption spectroscopy investigation into the crystal‐field transitions associated with Nd ions shifted toward shorter wavelengths. For transition, peak 4F_9/2_ shifted from 676.99 nm for the pure sample (*x* = 0) to 675.44 nm for the doped sample (*x* = 0.5). This study shows the correlation between bond characteristics and crystal‐field splitting. It also provides a method for fine‐tuning the crystal‐field energy by manipulating the electrostatic interactions in NdAlO_3_, along with providing the methodology for fine‐tuning the wavelength of laser radiation through adjustments in crystal‐field energy.

## Author Contributions

**Payal Ratnawat**: scientific discussion. **Ansh Dabkara**: scientific discussion and modelling. **Dibya Prakash Kar** scientific discussion. **Nikita Jain**: scientific discussion. **Minal Gupta**: formal analysis and optical spectroscopy measurements. **Archna Sagdeo**: X‐ray diffraction measurements and structural analysis. **Pankaj R. Sagdeo**: conceptualization and identification of the scientific problem, manuscript corrections, fruitful scientific discussions, and supervision of scientific work. **Akash S. Padole** and **Vikash Singar** contributed equally to this work.

## Conflict of Interest

The authors declare no conflict of interest.

## Data Availability

The data that support the findings of this study are available from the corresponding author upon reasonable request.

## References

[1] H. Zhang , N. Li , K. Li , D. Xue , Acta Crystallogr. B 2007, 63, 812. 18004035 10.1107/S0108768107046174

[2] E. A. R. Assirey , Saudi Pharm. J. 2019, 27, 817. 31516324 10.1016/j.jsps.2019.05.003 PMC6733782

[3] M. Bilal Hanif , M. Motola , S. Qayyum , S. Rauf , A. Khalid , C.‐J. Li , C.‐X. Li , Chem. Eng. J. 2022, 428, 132603.

[4] G. Pilania , P. V. Balachandran , J. E. Gubernatis , T. Lookman , Acta Crystallogr. Sect. B Struct. Sci. Cryst. Eng. Mater. 2015, 71, 507. 10.1107/S2052520615013979 26428400

[5] L. Zhu , R. Ran , M. Tadé , W. Wang , Z. Shao , Asia‐Pac. J. Chem. Eng. 2016, 11, 338.

[6] J. Peña‐Martínez , D. Marrero‐López , J. C. Ruiz‐Morales , B. E. Buergler , P. Núñez , L. J. Gauckler , J. Power Sources 2006, 159, 914.

[7] M. Kh. Hamad , E. Martinez‐Teran , Y. Maswadeh , R. Hamad , E. G. Al‐Nahari , A. A. El‐Gendy , Kh. A. Ziq , J. Magn. Magn. Mater. 2020, 514, 167171.

[8] A. Sati , P. Pokhriyal , A. Kumar , S. Anwar , A. Sagdeo , N. P. Lalla , P. R. Sagdeo , J. Phys. Condens. Matter 2021, 33, 165403. 10.1088/1361-648X/abf0bf 33752190

[9] A. Idrees , X. Jiang , G. Liu , H. Luo , G. Jia , Q. Zhang , L. Jiang , X. Li , B. Xu , ChemistryOpen 2018, 7, 688. 30191093 10.1002/open.201800097 PMC6121126

[10] M. Harilal , V. M. Nair , P. R. S. Wariar , K. P. Padmasree , M. M. Yusoff , R. Jose , Mater. Charact. 2014, 90, 7.

[11] C.‐F. Tseng , P.‐A. Lin , T.‐C. Wei , Jpn. J. Appl. Phys. 2015, 55, 01AA07.

[12] D. Petrov , B. Angelov , J. Sol‐Gel Sci. Technol. 2010, 53, 227.

[13] S. Mathur , M. Veith , H. Shen , S. Hüfner , M. H. Jilavi , Chem. Mater. 2002, 14, 568.

[14] M. Veith , S. Mathur , H. Shen , N. Lecerf , S. Hüfner , M. H. Jilavi , Chem. Mater. 2001, 13, 4041.

[15] A. Najafi , F. Sharifi , S. Mesgari‐Abbasi , G. Khalaj , Ceram. Int. 2022, 48, 26725.

[16] G. Khalaj , F. Soleymani , F. Sharifi , A. Najafi , J. Mater. Res. Technol. 2023, 26, 6182.

[17] Z. Mahmoudi , F. Soleymani , S. Mesgari Abbasi , G. Khalaj , A. Najafi , Ceram. Int. 2024, 50, 18081.

[18] F. Sharifi , Z. Mahmoodi , S. M. Abbasi , A. Najafi , G. Khalaj , J. Mater. Res. Technol. 2023, 22, 2462.

[19] M. Morshed , A. Najafi , G. Khalaj , Phys. Scr. 2024, 99, 035966.

[20] U. G. Akpan , B. H. Hameed , Appl. Catal. Gen. 2010, 375, 1.

[21] D. J. Newman , B. Ng , Crystal Field Handbook (Eds: D. J. Newman , B. Ng ), Cambridge University Press, Cambridge 2000, pp. 83–119, 10.1017/CBO9780511524295.007.

[22] R. G. Burns , Mineralogical Applications of Crystal Field Theory, 2nd ed., Cambridge Topics in Mineral Physics and Chemistry, Cambridge University Press, Cambridge 1993. 10.1017/CBO9780511524899.

[23] J. Yu , L. Cui , H. He , S. Yan , Y. Hu , H. Wu , J. Rare Earths 2014, 32, 1.

[24] P. Fulde , M. Loewenhaupt , Adv. Phys. 1985, 34, 589.

[25] M. Gupta , O. V. Rambadey , P. R. Sagdeo , Ceram. Int. 2022, 48, 23072.

[26] V. Grasso , F. Neri , P. Perillo , L. Silipigni , M. Piacentini , Phys. Rev. B 1991, 44, 11060. 10.1103/physrevb.44.11060 9999224

[27] J. B. Gruber , M. E. Hills , T. H. Allik , C. K. Jayasankar , J. R. Quagliano , F. S. Richardson , Phys. Rev. B 1990, 41, 7999. 10.1103/physrevb.41.7999 9993118

[28] J. Rodríguez‐Carvajal , Phys. B: Condens. Matter 1993, 192, 55.

[29] H. M. Rietveld , J. Appl. Crystallogr. 1969, 2, 65.

[30] K. Momma , F. Izumi , J. Appl. Crystallogr. 2011, 44, 1272.

[31] M. Gupta , S. Shirbhate , P. Ojha , S. Acharya , Solid State Ion. 2018, 320, 199.

[32] M. Gupta , A. Kumar , A. Sagdeo , P. R. Sagdeo , J. Phys. Chem. C 2021, 125, 2648.

[33] A. E. Danks , S. R. Hall , Z. Schnepp , Mater. Horiz. 2016, 3, 91.

[34] D. Navas , S. Fuentes , A. Castro‐Alvarez , E. Chavez‐Angel , Gels 2021, 7, 275. 34940335 10.3390/gels7040275 PMC8700921

[35] A. Kumar , M. K. Warshi , V. Mishra , A. Sati , S. Banik , A. Sagdeo , R. Kumar , P. R. Sagdeo , Ceram. Int. 2019, 45, 8585. 10.1088/1361-648X/ab1195 30893657

[36] R. D. Shannon , Acta Crystallogr. A 1976, 32, 751.

[37] R. Tilley , Perovskites: Structure‐Property Relationships, John Wiley & Sons, Ltd., Hoboken, NJ 2016, p. 315, 10.1002/9781118935651.

[38] P. M. Woodward , Acta Crystallogr. B 1997, 53, 44.

[39] N. Zettili , Quantum Mechanics: Concepts and Applications, John Wiley & Sons, Hoboken, NJ 2009.

[40] V. B. Pawade , V. Chopra , S. J. Dhoble , Spectroscopy of Lanthanide Doped Oxide Materials, Elsevier, Amsterdam 2020, pp. 1–20, 10.1016/B978-0-08-102935-0.00001-0.

[41] M. Hatanaka , S. Yabushita , J. Phys. Chem. A 2009, 113, 12615. 19746893 10.1021/jp9049507

[42] V. E. Fleischauer , G. Ganguly , D. H. Woen , N. J. Wolford , W. J. Evans , J. Autschbach , M. L. Neidig , Organometallics 2019, 38, 3124.

[43] L. Ungur , L. F. Chibotaru , Chem. – Eur. J. 2017, 23, 3708. 27983776 10.1002/chem.201605102

[44] M. Karbowiak , N. M. Edelstein , J. Drożdżyński , K. Kossowski , Chem. Phys. 2002, 277, 361.

[45] C. Görller‐Walrand , K. Binnemans , Handbook on the Physics and Chemistry of Rare Earths, Vol. 23, Elsevier, Amsterdam 1996, pp. 121–283, 10.1016/S0168-1273(96)23006-5.

[46] J. Gao , Q. Zhang , D. Sun , J. Luo , W. Liu , S. Yin , Opt. Commun. 2012, 285, 4420.

